# Supplementary material for: Psychometric properties of the Arabic version of the Existence Scale
Source: PLoS One. 2022 Apr 18;17(4):e0267321. doi: 10.1371/journal.pone.0267321 (PMC9015151; doi:10.1371/journal.pone.0267321)
Supplement: Attachment — Submitted filename: Response to Reviewers.docx [file pone.0267321.s002.docx]

**Response to Reviewers**

Dear editor,

We would like to thank you and for your valuable comments and feedbacks. Point-by-point responses to reviewers are listed below.

**Journal Requirements:**

Comment 1:

Please review your reference list to ensure that it is complete and correct. If you have cited papers that have been retracted, please include the rationale for doing so in the manuscript text, or remove these references and replace them with relevant current references. Any changes to the reference list should be mentioned in the rebuttal letter that accompanies your revised manuscript. If you need to cite a retracted article, indicate the article’s retracted status in the References list and also include a citation and full reference for the retraction notice.

Response 1:

Thank you for your valuable comments. We make sure that all our references are complete and correct.

Comment 2:

Additional Editor Comments:

Please clarify in the Methods that you were allowed to omit or modify the original questionnaire with permission form the developers (please include names).

Response 2:

Thank you for your valuable comments. We added a new statement regarding the permission from Alfried Längle to translate, omit, and modify the existence scale as follows:

“The official permission was sought from the developer (Alfried Längle) to translate, modify, and omit the ES as needed”.

**We hope now that our revised manuscript is acceptable for publication.**
